# Genetic Polymorphism in TNF-α-308 G/A and TNF-β +252 A/G, as Prognostic Biomarker in Breast Cancer Patients among Indian Population 

**DOI:** 10.31557/APJCP.2020.21.2.301

**Published:** 2020

**Authors:** Mohammad Margoob Ahmad, Farah Parveen, Naseem Akhter, Jamshaid Ahmad Siddiqui, Nootan Kumar Shukla, Syed Akhtar Husain

**Affiliations:** 1 *Department of Biotechnology, *; 2 *Department of Biosciences, Jamia Millia Islamia,*; 3 *Department of Transplant Immunology and Immunogenetics, *; 4 *Department of Surgical Oncology, BRA IRCH, All India Institute of Medical Sciences, New Delhi, India. *

**Keywords:** Cytokine, Immune responses, Single nucleotide polymorphisms (SNPs), Tumour Necrosis Factor (TNF)

## Abstract

**Background:**

Cytokines are the key regulator molecules that modulate immune response. Tumor necrosis factor (TNF- α-308 G/A and TNF-β +252 A/G ) are inflammatory cytokine that control the progression of several types of cancer. They play a vital role in both tumor progression and destruction based on their concentrations. The role of TNF-α-308 G/A and TNF-β +252 A/G gene polymorphism in the etiology of breast cancer (BC) is not clearly understood. Therefore, present study investigates the association of TNF-α -308 G/A and TNF-β +252 A/G and the clinical features with Breast cancer patients.

**Methods:**

In a case- control study, we have investigated 150 breast cancer patients and 300 age and ethnically matched healthy controls for duration of 3 years from North India. Promoter polymorphisms of tumor necrosis factor gene (TNF-α -308 G/A and TNF-β +252 A/G) were genotyped using allele specific oligonucleotide polymerase chain reaction ASO and restriction fragment length polymorphism (PCR-RFLP). The associations were evaluated by calculating the pooled odds ratio (OR) with 95% confidence interval (95% CI) using SPSS.

**Results:**

Patients with different clinico-pathological variables and healthy controls were analyzed. Significant association was observed in A allele of TNF-α -308 G/A in breast cancer patients as compared to healthy controls (p<0.0001). However, no association was seen in TNF-β +252 A/G both at genotypic and allelic level. The GG genotype of TNF-β +252A/G is higher in grades III (p<0.01) patients.

**Conclusion:**

Our results suggest that TNF-α-308G/A polymorphism showed significant association with breast cancer patients.

## Introduction

Breast cancer is one of the major cancers affecting the mortality of women worldwide (Jemal et al., 2008). It is second most common cancer, with a prediction of increase in new cases upto 22 million in two decades (Ferlay et al., 2012). Various important risk factors reported for breast cancer are long time fertility, use of oral contraceptives, nulliparity, obesity post menopause, hormone replacement therapy, various genetical, environmental and life style factors (Torre et al., 2012). Breast carcinogenesis occurs because of the complex interactions between multiple environmental and genetic factors. However, the mechanisms of the carcinogenesis at the molecular level remain poorly understood. Genetic factors can serve as a susceptibility variable for breast cancer development, and their identification can help to reduce the incidence of breast cancer (Njiaju et al., 2012). 

Cytokines are key molecules that modulate immune response and play crucial role in cancer immunity (Smith et al., 2004). Tumour necrosis factor- α (TNF-α) is a multi-functional cytokine involved in promotion of inflammatory responses, cell proliferation, differentiation, apoptosis, lipid metabolism, coagulation, insulin resistance, malignant diseases and promotion of angiogenesis through the stimulation of endothelial cell proliferation (Bower et al., 2013; Leek et al., 1998). TNF-α is known to exhibit anti-tumor activities in a variety of tumor cell lines. It can be produced locally in the transformed mammary gland by tumor infiltrating lymphocytes or by cells of tumor stroma. TNF-α is also involved in the increased expression of adhesion molecule (such as VCAM-1 or P-selectin) on the endothelium of tumor vessels, leading to the necrosis of tumor (Champ et al., 2012; Loculano et al., 1995). TNF-α has a receptor on tumor cells that stimulates activation and prolonged expression of the JUN oncogene; participates in apoptosis; stimulates the hydrolysis of sphingomyelin, ceramide, and phosphorylcholine; and induces the production of reactive oxygen species (Cereda et al., 2012). Association of elevated plasma levels of TNF-α have been discovered in many malignancies with poor prognoses (Cereda et al., 2012; Nakashima et al., 1998; Szlosarek et al., 2003). Knockdown of the *TNF-α* gene is associated with cell proliferation inhibition and apoptosis in TNBC (Pileczki et al., 2012).

Various single nucleotide polymorphisms (SNPs) are found in the TNF-α promoter regions, which can regulate the expression level of *TNF-α*, such as *TNF-α-308G/A* (*rs1800629*) and *TNF-β 252 AG* (*rs361525*) (Fargion et al., 2001). Out of these *SNPs TNF -α-308G/A *(*rs1800629*) and *TNF-β -252 A/G* (*rs361525*) polymorphism is the most studied (Kroeger et al., 1997). TNF-α 308G/A is a G to A transition at nucleotide position 308 in the promoter region of the gene and TNF-β 252 A/G is a A to G transition at nucleotide position 252 in the promoter region of the gene. It has been shown in previous studies that polymorphisms of TNF-α at positions 308 and 238 (i;e TNF2 and TNFA alleles) are associated with increased release of *TNF-α *(D’Alfonso et al.,1994; Kroeger et al.,1997).

The role of *TNF-α *is important in cancer development therefore common functional polymorphisms (rs1800629 and rs361525) have been examined extensively in many studies. The polymorphism of *TNF-α (-308 G/A) *have been correlated with the low (G) and high (A) production of this cytokine (Perry et al., 1998). Studies have illustrated the plasma level of TNF-α or β is associated with the outcome of certain solid and hematological malignancies (Dosquet et al., 1997; Kobayashi et al., 1997). The anti-tumor and anti-metastatic effect of TNF-β on human solid tumors transplanted is also shown in mice (Funahashi et al., 1991; Qin et al., 1995). Many studies have explained the association between cytokine polymorphism and development of different cancers including chronic lymphocytic leukemia, non-Hodgkin lymphoma and breast cancer (Howell et al., 2007). However still there are conflicting results showing susceptibility (Chouchane et al., 1997) and no susceptibility to the breast cancer disease (Gaudet et al., 2007).

Present study was designed to investigate the association of *TNF -α-308G/A (rs1800629)* and *TNF-β +252 A/G (rs361525)* gene and their association with breast cancer in Indian population. In present case-control study we have also tried to correlate the role of TNF-α -308G /A and TNF-β +252 A/G in Breast Cancer susceptibility by ER, PR and Her2 status and the relationship between genotypes and clinicopathological characteristics of Breast Cancer. 

## Materials and Methods

*Case Control Study*

Patients and Control: The breast cancer patients ranged between 20 and 80 years of age attending the out patient department for treatment in DR. B. R. Ambedkar-Institute Rotary Cancer Hospital (BRA-IRCH) and All India Institute of Medical Sciences (AIIMS), New Delhi (India) from July 2011 till June 2013 were taken as cases. This was a case control study for the duration of 3 years. The controls were normal healthy adults (between18 and 40 years of age) without any history of malignancy or any other disease. 

*Ethical Statement*

The study was performed as per the ethical standards laid down by the Declaration of Helsinki. The study was approved by ethics committee of Jamia Millia Islamia (A central university) and All India Institute of Medical Sciences, New Delhi, India. Written informed consent from all the patients and healthy controls was obtained before registering them for the study.

Inclusion and Exclusion Criteria: A total of 150 blood samples from genetically unrelated women with sporadic breast cancer cases and and 300 healthy controls without disease or family history of breast cancer were included in the study. Inclusion criteria included female breast cancer patients in the age group 20 to 79 years with life expectancy of atleast 6 months, histopathological confirmation with primary breast cancer and patients ready to consent and abide by the trial related procedures. Exclusion criteria included in the study were previous exposure to chemotherapy or radiotherapy, patients with multiple cancers or pregnant women and patients with acute myocardial or surgical complications.

*Genomic DNA samples*

All the samples were collected from patients and healthy controls of the same ethnic group from Indian population. All the subjects included in this study lived in Delhi- NCR provice of India. A total of 150 blood samples were collected from the breast cancer patients and 300 healthy controls without disease or family history of breast cancer. 

*Genotype Analysis*

Peripheral blood samples (5ml) were collected in EDTA-coated vials and stored at 20^o^C until use. The genomic DNA was extracted from whole blood by the salting out procedure (Miller et al., 1998). The differential production of cytokine is caused by single nucleotide polymorphism (SNPs) in the promoter, coding, non-coding regions of cytokine genes. The SNPs including *TNF-α *(*-308 G/A*), *TNF-β *(*+252 A/G*) were determined in female breast cancer patients and healthy controls. The genotyping was performed using allele specific oligonucleotide polymerase chain reaction (ASO-PCR) for *TNF-308* (*rs1800629G/A*) using primers 5’-TCCTGCATCCTGTCTGGAA-3’ (forward), 5’-AGCGGAAAACTTCCTTGGT-3’(reverse) and restriction fragment length polymorphism (RFLP) for TNF-β (+252 A/G) using the primers F: 5’-CCGTGCTTCGTGGTTTGGACT-3’ R: 5’ -AGAGGGGTGGATGCTTGGGTTC-3’. 

The genotyping of *TNF-α 308 G/A* (*rs1800629*) was performed by ASO-PCR and the 100 bp amplified product of β-globulin was used as internal control. The total 25µl PCR reaction mixture consists of 500 ng of genomic DNA, 200 µmol/l dNTPs, 2 mM MgCl_2_, 1XTaq DNA polymerase buffer, 2 units of Taq DNA polymerase (Merk India),10 pmol of each test primer and 5 pmol of internal control primers. The thermal condition for PCR include 95^o^C for 5 min; 31 cycles of 94^o^C for 30 sec, 61^o^C for 150 sec, and 72^o^C for 30 sec; and final extension of 72^o^C for 10 min. The amplified product from each well was performed gel electrophoresis using 2.0% agarose and stained with ethidium bromide. 

The TNF–β +252A/G (rs361525) was genotyped using the PCR-RFLP method. The thermal condition includes 95^o^C for 5 min; 31 cycles of 94^o^C for 30 sec, 61^o^C for 150 sec, and 72^o^C for 30sec; and final extension of 72^o^C for 10 min. The amplified product of TNF-β +252A/G was digested with 5 units Nco-I (Fermantas) at 37^o^C for 16 hrs. The undigested single fragment was of 782 bp and digested one give two 586 bp and 196 bp reaction products that were separated on 2% agarose gel electrophoresis. 

*Statistical analysis*

The sample size was calculated prior to the study using Quanto software version 1.2. Allele and genotype frequencies were determined by PopGen v16. Frequency differences between the Breast cancer and control groups were tested for signiﬁcance using Fisher’s exact test with Bonferroni correction. The magnitude of the effect was estimated by odds ratios and their 95% conﬁdence intervals (Windows 11.0.0.2001; SPSS Inc.). Logistic regression was used to test the association between the variant as well as wild-type genotypes and the prevalence of breast cancer. P-values less than or equal to 0.05 were considered significant. 

## Results

The majority of 150 breast cancer cases recruited for the study was sporodic 131 (87.3 % ) while familial cases found were 19 (12.7%). The age of patients diagnosed with breast cancer ranged from (20-89) years . The majority of the cases were in the age group of 40-49 years, and age of the patients was calculated. 56 (37.3%) cases were pre-menopausal while 94 (62.7%), attained the menopausal. Therefore, according to the mensutral staus, the incidence of breast carcinoma was found more in post-menopausal women. The 147 (98%) were infiltrating ductal carcinoma and 3 (2%) were infiltrating lobular carcinoma of the breast cancer patients. The majority of the patients had infiltrating ductal carcinoma. The Clinical staging of the tumor was done according to AJCC (American joint committee on Cancer) recommendation. A total of patients 4 (2.7%) belong to stage I, 33 (22.0%) to stage IIa, 34 (22.7%) to IIb, 40 (26.7%) to IIIa, 24 (16%) to IIIb, 10 (6.7%) to IIIc and 5 (3.4%) to IV. Out of 150 cases of breast carcinoma, only 66 (44%) had pathologically negative for axillary lymph nodes, while 84 (56%) cases had positive axillary nodes. Thus majority of the cases were having lymph node involvement. The total 36 (24%) were having >2cm tumor size and 114 (76%) were >2 cm tumor size of the breast cancer patients. Thus majority of the cases were having >2 cm tumor size. Estrogen receptor status was evaluated in all the patients recruited in the study. Out of total 150 patients 67(44.7%) were Estrogen receptor (ER) positive while, the rest of the patients 83 (55.3%) were ER- negative. Out of the total of 150 cases 71 (47.3%) were positive progestrone receptors and 79 (52.7%) were negative for progestrone. The total of 82 (54.7%) were positive HER-2/neu and 68 (45.3%) were negative HER-2/neu of the breast cancer patients.

*TNF-α 308 G/A (rs1800629) Gene Polymorphism*

The genotype and allele frequency data revealed that A allele of TNF-α 308 was found to be susceptible in nature. The GG genotype of TNF-α was significantly higher in the healthy controls as compared to patients (69% vs 45.3%, p<0.0001). Whereas, GA genotype was significantly higher in patients as compared to controls (Table 2). No significant association was observed for AA genotype when compared among patients and controls. The frequency of GG, GA and AA genotype were not significantly different in clinico-pathological grade including Pathological grades, Menstrual status, Estrogen receptor, Progesterone receptor, Herception receptor (HER-2/neu). On the other hand, the frequency of GG genotype was significantly associated between Lymph node status. The homozygous GG genotype in Lymph node positive (36.9%) was significantly lower as compared to Lymph node negative patients (56.0 %, p=0.02). 

*TNF-β +252A>G (rs361525) Gene Polymorphism*

The allelic and genotypic frequencies of TNF-β +252A/G were compared in the patients and healthy controls. No significant association of this cytokine was seen both at allelic and genotypic level in both the groups. The frequency of GG genotype (3.1%) in grade (I, II) was significantly lower as compared to grade III (17.4%) (p=0.019) (Table 3). The heterozygous GA genotype in pre-menopausal was significantly lower as compared to post-menopausal patients. The frequency of AA genotype in pre-menopausal (71.4%) was significantly higher than post-menopausal (48.9%, p=0.012) patients (Table 3). Similarly, no correlation was observed with lymph node status, presence of estrogen, progesterone, herception receptor (HER-2/neu) (Table 4). 

**Figure 1:**
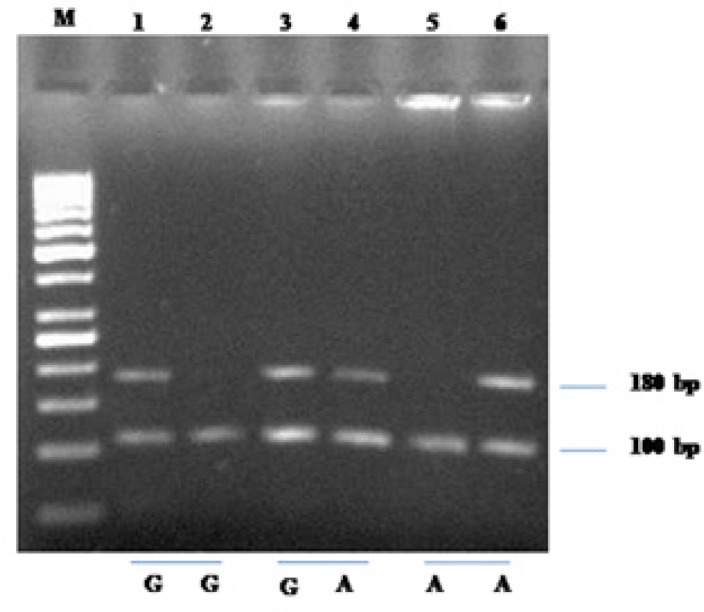
Representative Band Patterns of the PCR Products for TNF-α (-308G/A) (rs1800629) Polymorphism. M stands for molecular size marker of 50 bp ladder. Determination of TNF-α genotypes by ASO-PCR: two well (one for A specific primer, one for G specific primer) were used for on patient. 180 bp represent presence of A or G allele in respective well. 100 bp band represent internal control

**Table 1:** Clinco - Epidemilogical Features of the Breast Cancer Patients

Clinical–Epidemiological features	Cases (n= 150)	Percentage (%)
Familial breast Cancer	19	12.7
Sporadic breast cancer	131	87.3
Age group		
20-29	5	3.3
30-39	25	16.7
40-49	44	29.3
50-59	38	25.3
60-69	28	18.7
70-79	10	6.7
Menstrual status		
Pre- menopausal	56	37.3
Post- menopausal	94	62.7
Histological stage		
Infiltrating ductal carcinoma	147	98.0
Infltrating lobular carcinoma	3	2.0
Clinical staging		
I	4	27.0
IIa	33	22.0
IIb	34	22.7
IIIa	40	26.6
IIIb	24	16.0
IIIc	10	6.7
IV	5	3.3
Lymph node status		
Positive	84	56.0
Negative	66	44.0
Tumor size pathologic		
≤2cm	36	24.0
> 2cm	114	76.0
Estrogen receptor		
Positive	67	44.7
Negative	83	55.3
Progesterone receptor		
Positive	71	47.3
Negative	79	52.7
HER-2/neu		
Positive	82	54.7
Negative	68	45.3

**Table 2:** Genotype Analysis and Allele Frequencies of TNF-α and TNF-β Gene in Breast Cancer Patients and Healthy Controls

Genotype	Patients N=150	Control N=300	p-value	OR (95%CI)
TNF-α (-308) (rs1800629G>A)				
GG	68 (45.3)	207 (69.0)	<0.0001	0.37 (0.24-0.55)
GA	73 (48.6)	80 (26.6)	<0.0001	2.60 (1.73-3.92)
AA	9 (6.0)	13 (4.3)	0.4887	1.40 (0.58-3.37)
G Allele	209 (69.66)	494 (82.3)	<0.0001	0.49 (0.35-0.68)
A Allele	91 (30.33)	106 (17.66)	<0.0001	2.02 (1.46-2.80)
TNF-β (+252) (rs361525 A>G)				
AA	86 (57.3)	155 (38.33)	0.2712	1.25 (0.84-1.86)
AG	56 (37.3)	118 (39.33)	0.7581	0.91 (0.61-1.37)
GG	8 (5.3)	27 (9.0)	0.195	0.56 (0.25-1.28)
A Allele	228 (76)	428 (71.33)	0.1523	1.27 (0.92-1.75)
G Allele	72 (24)	172 (28.66)	0.1523	0.78 (0.57-1.08)

**Table 3:** Genotypic Frequencies TNF-α (-308) and TNF-β(+ 252), Genotype and Lymph Node Status, Pathological Grades Menstrual Status

Genotype	Total (n=150) (%)	Number of Lymph node positive (n=84) (%)	Number of lymph node negative (n=66) (%)	p value	OR (95%CI)
TNF-α (-308) (rs1800629G>A)					
GG	68 (45.3)	31 (36.9)	37 (56.0)	0.0217	0.45 (0.23-0.88)
GA	73 (48.6)	46 (54.7)	27 (40.9)	0.1022	1.74 (0.91-3.35)
AA	9 (6.0)	7 (8.3)	2 (3.0)	0.2996	2.90 (0.58-14.50)
TNF-β (+252) (rs361525 A>G)					
AA	86 (57.3)	46 (54.7)	40 (60.6)	0.5089	0.78 (0.40-1.51)
AG	56 (37.4)	33 (39.2)	23 (34.8)	0.613	1.21 (0.61-2.36)
GG	8 (5.3)	5 (5.9)	3 (4.5)	1	1.32 (0.30-5.77)
Genotype	Total (n=150) (%)	Number of Grade I & II (n=127) (%)	Number of Grade III (n=23) (%)	p value	OR (95%CI)
TNF-α (-308) (rs1800629G>A)					
GG	68 (45.3)	60 (47.2)	8 (34.7)	0.3633	1.67 (0.66-4.24)
GA	73 (48.6)	59 (46.4)	14 (60.8)	0.2585	0.55 (0.22-1.38)
AA	9 (6.0)	8 (6.2)	1 (4.3)	1	1.47 (0.17-12.42)
TNF-β (+252) (rs361525 A>G)					
AA	86 (57.3)	73 (57.4)	13 (56.5)	1	1.04 (0.42-2.54)
AG	56 (37.4)	50	6 (26.0)	0.2518	1.84 (0.67-4.98)
GG	8 (5.3)	4 (3.1)	4 (17.3)	0.0197	0.15 (0.03-0.67)
Genotype	Total (n=150) (%)	Pre- menopause (n=56) (%)	Post-menopause (n=94) (%)	P value	OR (95%CI)
TNF-α (-308) (rs1800629G>A)					
GG	68 (45.3)	23 (41.1)	45 (47.8)	0.4982	0.75 (0.38-1.48)
GA	73 (48.6)	29 (51.8)	44 (46.8)	0.6139	1.22 (0.62-2.36)
AA	9 (6.0)	4 (7.1)	5 (5.3)	0.7279	1.36 (0.35-5.32)
TNF-β (+252) (rs361525 A>G)					
AA	86 (57.3)	40 (71.4)	46 (48.9)	0.0102	2.60 (1.28-5.29)
AG	56 (37.4)	14 (25.0)	42 (44.7)	0.0228	0.41 (0.19-0.85)
GG	8 (5.3)	2 (3.6)	6 (6.4)	0.7196	0.54 (0.10-2.79)

**Table 4:** Genotypic Frequencies of TNF-α (-308) and TNF-β (+ 252), Genotype and Estrogen Receptor Status, Progesterone Receptor status, Herception Receptor Status

Genotype	Total (n=150) (%)	Estrogen Receptor Positive (n=67) (%)	Estrogen Receptor Negative (n=83) (%)	p value	OR(95%CI)
TNF-α (-308) (rs1800629G>A)					
GG	68 (45.3)	28 (41.7)	40 (48.1)	0.5099	0.77 (0.40-1.47)
GA	73 (48.6)	34 (50.7)	39 (46.9)	0.7428	1.16(0.61-2.21)
AA	9 (6.0)	5 (7.4)	4 (4.8)	0.5137	1.59(0.41-6.18)
TNF-β (+252) (rs361525 A>G)					
AA	86 (57.3)	38 (56.7)	48 (57.9)	1	0.95(0.49-1.83)
AG	56 (37.4)	27 (40.3)	29 (34.9)	0.6107	1.25 (0.64-2.44)
GG	8 (5.3)	2 (3.0)	6 (7.2)	0.2987	0.39 (0.07-2.02)
					
Genotype	Total (n=150) (%)	Progesterone Receptor Positive (n=71) (%)	Progesterone Receptor Negative (n=79) (%)	p value	OR(95%CI)
TNF-α (-308) (rs1800629G>A)					
GG	68 (45.3)	36 (50.7)	32 (40.5)	0.2511	1.51 (0.79-2.88)
GA	73 (48.6)	31 (43.6)	42 (53.1)	0.2571	0.68 (0.35-1.30)
AA	9 (6.0)	4 (5.6)	5 (6.3)	1	0.88 (0.22-3.42)
TNF-β (+252) (rs361525 A>G)					
AA	86 (57.3)	45 (63.4)	41 (52.0)	0.1868	1.60 (0.83-3.08)
AG	56 (37.4)	23 (32.4)	33 (41.7)	0.2432	0.66 (0.34-1.30)
GG	8 (5.3)	3 (4.2)	5 (6.3)	0.7223	0.65 (0.15-2.83)
Genotype	Total (n=150) (%)	Herceptin Receptor Positive (n=82) (%)	Herceptin Receptor Negative (n=68) (%)	p value	OR (95%CI)
TNF-α (-308) (rs1800629G>A)					
GG	68 (45.3)	37 (45.1)	31 (45.5)	1	0.98 (0.51-1.87)
GA	73 (48.6)	41 (50)	32 (47.0)	0.7451	1.12 (0.59-2.14)
AA	9 (6.0)	4 (4.9)	5 (7.3)	0.7321	0.64 (0.16-2.50)
TNF-β (+252) (rs361525 A>G)					
AA	86 (57.3)	49 (59.8)	37 (54.4)	0.6191	1.24 (0.64-2.38)
AG	56 (37.4)	28 (34.1)	28 (41.2)	0.4005	0.74 (0.38-1.44)
GG	8 (5.3)	5 (6.1)	3 (4.4)	0.7291	1.40 (0.32-6.11)

**Figure 2:**
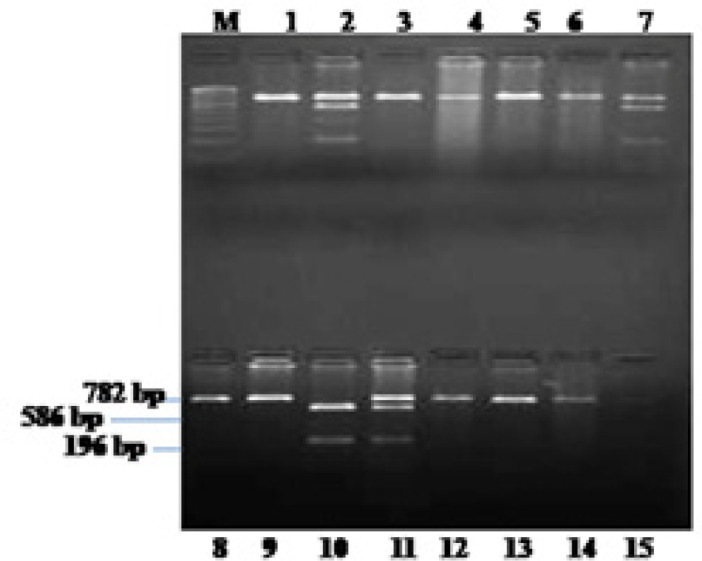
Representative Band Patterns of the PCR Products for TNF-β-252 (rs361525) Gene Polymorphism. M stands for molecular size marker of 100 bp ladder. Determination of TNF-β genotypes by PCR-RFLP: Sample no. 1 represents AA genotype (782 bp band), sample no.2 represent AG genotype (782, 586 and 196 bp), sample no. 10 represent GG genotype (586, 196 bp).

## Discussion

In the present study, we have attempted to examine *TNF-a 308G/A (rs1800629)* and *TNF-β +252 A/G (rs361525)* gene polymorphism both at genotypic and allelic level and their association with breast cancer patients and healthy controls in Indian population. We provided evidence that patients with TNF-α -308A allele had an increased risk of tumor metastasis. Our study highlights the effect of the TNF-α 308 gene polymorphism on the progression of BC.

Cytokine genes responsible for inflammation and metabolism have received special attention in cancer genetics. Among malignancies the association of cytokine genes and breast cancer are the best studied and the association of TNF polymorphisms has received special interest. Many studies have been conducted to understand the role of genetic polymorphism in cytokine genes including *TNF-α *and *TNF-β *in breast cancer patients (Saha et al., 2003; Balasubramanian et al., 2006). The findings of our study were equivalent to those reported by other researchers who demonstrated the association of the *TNF- α 308* gene polymorphism with breast cancer (Park et al., 2002).

TNF-α 308G/A promoter polymorphism affects the expression of TNF- α. A ‘guanine’ at this position is associated with low TNF-α production, whereas an ‘adenine’ with the high production therefore increases breast cancer risk (Wilson et al., 1997 ; Perry et al., 1998). Recently a study conducted on USA population reported that *TNF-α 308G/A* polymorphism was associated with reduced breast-cancer-specific and all-cause mortality (Duggan et al., 2017). Similarly, other studies conducted on Caucasian (Azmy et al., 2004), Iranian, Turkish (Gonullu et al., 2007), Korean (Mestiri et al., 2001) populations have not shown any correlation with *TNF-α -308G/A* and breast cancer disease. However, genetic polymorphism of TNF and IL-6 cytokines did not play a significant role to the cytokine fluctuations as well as cognitive impairment in Asian cohort (Chae et al., 2016). 

In contrast, a study on Tunisian population demonstrated a positive association between the *TNF-α (-308 G/A) *polymorphism and breast cancer susceptibility (Chouchane et al., 1997). Our study has shown that GA genotype is significantly associated with the development of breast cancer disease. However, GG genotype which is a low producer of TNF-α is associated with protection from the development of disease. Like our findings where the ‘A’ allele frequency was lower another study also showed lower frequency in Iranian population than Tunisian population (Mestiri et al., 2001). The distribution of TNF-α genotypes were not associated with the nodal status and tumor grade which were in accordance with another investigator (Azmy et al., 2004; Eskandar Kamali-Sarvestani et al., (2005). Two meta analysis also reported that GA and AA genotypes of TNF-α-308 were significantly associated with decreased BC risk in Caucasians (Yang et al., 2011; Wang et al., 2011). The menopausal status showed that the GA and AA, genotype was significantly associated with the patients with pre-menopausal group. The GA genotype is significantly associated with the development of disease in post-menopausal patients as compared to pre-menopausal. Steroid receptors such as estrogen receptor (ER) and progesterone receptor (PR) are also responsible for the regulation of genes involved in controlling cell growth. In fact, most breast tumors are initially dependent upon estrogen to support their growth. 

Our results have not shown any significant impact of TNF-α genotypes with the presence of Estrogen and progesterone receptor which were in accordance with (Kamali-Sarvestani et al., 2005 ) where allelic distribution of the TNF-α is not significantly associated with Estrogen and Progesterone receptor expression. We have also not observed any significant association of HER-2/Neu status and the breast cancer disease and its progression. 

The *TNF-β (+252 A/G)* gene polymorphism conducted in different population/ethnicity compared the cases with control, demonstrate conflicting results (Gonullu et al., 2007). Park et al., (2002) have shown that the AA genotype of TNF-β (+252 A/G) position conferred resistance for the breast cancer in Korean population. However, there are conflicting results where it has been reported that no significant differences were observed in allele and genotypic frequencies of TNF-β in breast cancer patients in population of Iran and Poland (Kamali-Sarvestani et al., 2005; Dosquet et al., 1997). Another study from India explains TNF-β intron 1 +252 A/G (rs909253) in two ethnically different population groups from Indo-European (North Indian), Dravidian (South Indian) groups. The* TNF-β *polymorphism seems to display its association in an ethno-specific manner. The major and minor allele frequency in Indo-European group does not associate with breast cancer risk while it associates strongly in Dravidian group (Pooja et al., 2011; Kohaar et al., 2009). Based on meta-analysis, Zhou et al., (2012) reported no significant association between TNF-252-β A/G and breast cancer, although positive association was found in Asian populations. But, in our study also we have not observed any significant association of TNF-β genotype between breast cancer cases and controls group. Further, the analysis of nodal status positive and negative group also did not reveal any significant associations which were in accordance with other study (Kamali-Sarvestani et al., 2005). However, a significant association was found for AA genotype of TNF-β in pre-menopausal status of the patients. Similarly, lee et al reported that the risk of menopausal breast cancer increased in parallel the number of the genotypes risk in Korean population (Lee et al., 2005). Interestingly, we have not found any correlation with between these genotypes with the presence of estrogen, progesterone and Her2/Neu receptor. The main limitation of our study is small sample size which could not detect the susceptibility of *TNF-α-308G/A* polymorphism in each subtypes of breast cancer. The functional study was not performed to determine the role of TNF-α-308G/A and TNF-β +252 A/G. 

In conclusion, the data of the present study indicate that TNF-α gene polymorphism is associated with breast cancer disease. The GA genotype is also significantly associated with post-menopausal category of patients. The result may be utilized in risk stratification and predictive treatment outcome in these categories of patients. Next, the GG genotype of TNF-β was predictive in grade 3 category of patients. Also, the AA genotype of this cytokine is associated with patients in the pre-menopausal stage. So, different SNPs may serve as biomarkers in various categories of patients. These may be utilized in disease prognosis, differential treatment modality and disease outcome. This study highlights the role of genetic biomarkers that are not changing with time. The results may be used by the clinicians in advance molecular medicine-based treatment in future. More extensive studies on multiple centers and ethnic populations may illustrate clear clinical utilization and this may be opted as biomarkers for best patient treatment. 
